# Electroencephalographic features in patients undergoing extracorporeal membrane oxygenation

**DOI:** 10.1186/s13054-020-03353-z

**Published:** 2020-10-30

**Authors:** Lorenzo Peluso, Serena Rechichi, Federico Franchi, Selene Pozzebon, Sabino Scolletta, Alexandre Brasseur, Benjamin Legros, Jean-Louis Vincent, Jacques Creteur, Nicolas Gaspard, Fabio Silvio Taccone

**Affiliations:** 1 Department of Intensive Care, Erasme Hospital, Université Libre de Bruxelles (ULB), Route de Lennik, 808, 1070 Brussels, Belgium; 2 Department of Medical Biotechnologies, Anesthesia and Intensive Care Unit, University of Siena, Via Bracci 1, 53100 Siena, Italy; 3 Department of Neurology Erasme Hospital, Université Libre de Bruxelles, Route de Lennik, 808, 1070 Brussels, Belgium; 4 Department of Neurology, Yale University Medical School, 15, York Street, New Haven, CT 06510 USA

**Keywords:** ECMO, Electroencephalogram, EEG, Outcome, Neurological complication, Cardiac arrest

## Abstract

**Background:**

Neurologic injury is one of the most frequent causes of death in patients undergoing extracorporeal membrane oxygenation (ECMO). As neurological examination is often unreliable in sedated patients, additional neuromonitoring is needed. However, the value of electroencephalogram (EEG) in adult ECMO patients has not been well assessed. Therefore, the aim of this study was to assess the occurrence of electroencephalographic abnormalities in patients treated with extracorporeal membrane oxygenation (ECMO) and their association with 3-month neurologic outcome.

**Methods:**

Retrospective analysis of all patients undergoing venous–venous (V–V) or venous–arterial (V–A) ECMO with a concomitant EEG recording (April 2009–December 2018), either recorded intermittently or continuously. EEG background was classified into four categories: mild/moderate encephalopathy (i.e., mostly defined by the presence of reactivity), severe encephalopathy (mostly defined by the absence of reactivity), burst-suppression (BS) and suppressed background. Epileptiform activity (i.e., ictal EEG pattern, sporadic epileptiform discharges or periodic discharges) and asymmetry were also reported. EEG findings were analyzed according to unfavorable neurological outcome (UO, defined as Glasgow Outcome Scale < 4) at 3 months after discharge.

**Results:**

A total of 139 patients (54 [41–62] years; 60 (43%) male gender) out of 596 met the inclusion criteria and were analyzed. Veno–arterial (V–A) ECMO was used in 98 (71%); UO occurred in 99 (71%) patients. Continuous EEG was performed in 113 (81%) patients. The analysis of EEG background showed that 29 (21%) patients had severe encephalopathy, 4 (3%) had BS and 19 (14%) a suppressed background. In addition, 11 (8%) of patients had seizures or status epilepticus, 10 (7%) had generalized periodic discharges or lateralized periodic discharges, and 27 (19%) had asymmetry on EEG. In the multivariate analysis, the occurrence of ischemic stroke or intracranial hemorrhage (OR 4.57 [1.25–16.74]; *p* = 0.02) and a suppressed background (OR 10.08 [1.24–82.20]; *p* = 0.03) were independently associated with UO. After an adjustment for covariates, an increasing probability for UO was observed with more severe EEG background categories.

**Conclusions:**

In adult patients treated with ECMO, EEG can identify patients with a high likelihood of poor outcome. In particular, suppressed background was independently associated with unfavorable neurological outcome.

## Background

In the last decade, the number of patients treated with extracorporeal membrane oxygenation (ECMO) for cardiopulmonary and respiratory failure has significantly increased [1, 2]. The use of ECMO can lead to a number of major complications [3]. Among them, neurological complications, such as ischemic and hemorrhagic stroke and seizures, are associated with longer hospital stay and increased risk of poor outcome [4]. Several studies documented a high occurrence of neurological complications during ECMO treatment among infants and children, while more recent studies described such complications also in adult patients [4–6]. As patients undergoing ECMO support usually requires sedation, neurological examination is not always reliable and neuromonitoring becomes important to detect rapidly such neurological complications. There has been increasing interest in the application of different neuromonitoring tools in ECMO patients, including electroencephalography (EEG), neuroimaging, near-infrared spectroscopy (NIRS) and chemical biomarkers [7]; the available data on their usefulness in adult ECMO patients remain scarce [8].

EEG is a noninvasive tool that measures cortical electrical activity, with a good spatial and temporal resolution and sensitivity to changes in both brain structure and function [9]. In critically ill patients, EEG is widely used to recognize or assess different neurological complications, such as seizure and encephalopathy [10, 11], or to prognosticate neurological outcome after an acute brain injury [12, 13]. Nevertheless, the use of EEG as neuromonitoring tool in adult ECMO patients has also been reported only in small case-series [8, 14].

The aim of this study was therefore to evaluate the occurrence of EEG abnormalities and their prognostic value in adult ECMO patients.

## Methods

### Study design and patient selection

This retrospective study was performed in the Department of Intensive Care at Erasme Hospital, Brussels (Belgium). The local Ethical Committee (Comité d’Ethique Hospitalo-Facultaire Erasme-ULB) approved the study but waived the need for informed consent because of its retrospective nature (Protocol 2018/263). From our institutional database (April 2009–December 2018), all patients undergoing veno–arterial (V–A) and veno–venous (V–V) ECMO and who had a concomitant EEG monitoring were considered eligible for the study. EEG was recorded either as continuous or discontinuous. Exclusion criteria were: ECMO missing data; EEG artifacts that made EEG records unreadable; death before 24 h from ICU admission, as the occurrence of neurological dysfunction could not be evaluated and EEG recording was quite limited.

### Patients’ care and monitoring

All patients were monitored using an arterial catheter and a central venous catheter. A deep sedation status during mechanical ventilation was initially obtained using a combination of sedatives (i.e., midazolam or propofol) and analgesics (i.e., morphine or sufentanil) and then adjusted according to clinical needs. Advanced hemodynamic monitoring was used (PiCCO, Pulsion, Munich, Germany) and the assessment of cardiac function by repeated trans-esophageal and/or trans-thoracic echocardiography. Mean arterial pressure was maintained at > 65–70 mmHg using volume resuscitation, noradrenaline and/or dobutamine, whenever needed, or by adjusting the ECMO blood flow in veno-arterial (V-A) configuration. Blood flow, FiO_2_ and gas flow of the ECMO were adapted to maintain PaO_2_ between 60 and 150 mmHg and PaCO_2_ between 35 and 45 mmHg, with a prior adaptation of the ventilator for a protective ventilation associated to the lowest FiO_2_. Blood glucose was kept between 110 and 150 mg/dl using continuous insulin infusion. Enteral nutrition was initiated as soon as possible and continued thereafter according to gastric tolerance. Management of ECMO is reported in Additional file 1.

### Data collection and definition

We collected data on demographics, comorbidities, indications and duration of ECMO support, as well as ICU length of stay and hospital mortality. We also collected Glasgow Coma Score during ECMO and major complications during ECMO therapy (i.e., cerebral ischemic stroke; intracranial hemorrhage—ICH), brain death, major bleeding, i.e., reduction of at least 2 g/dL in hemoglobin levels requiring ≥ 4 RBC units to be transfused over 24 h), ECMO configuration, the use of red blood cells transfusions (RBCT), sedative, analgesic and/or antiepileptic drugs. Also, the results from brain imaging were collected, whenever available during the entire ICU stay. We also recorded mechanical ventilation settings and ECMO parameters for the first 2 days of EEG monitoring. For the final analysis, only the worst of these values were considered.

Neurological evaluation at 3 months after ECMO insertion was assessed using the Glasgow Outcome Scale (GOS; 1 = Death or severe injury without recovery of consciousness; 2 = Persistent vegetative state; 3 = Severe injury with permanent need for help with daily living; 4 = Moderate disability with no need for assistance in everyday life; 5 = Low disability with minor neurological and psychological deficits) [15]. The GOS evaluation was assessed during follow-up visits or by telephone interview with the general practitioner [16]. Favorable neurological outcome (FO) was considered as a GOS 4–5, unfavorable outcome (UO) as GOS 1–3.

### EEG recording and definition

The EEG recording was started by medical indication and was either discontinuous (i.e., 30 min) or continuous (i.e., more than 12 h), depending on clinical patient’s status and medical decisions. Twenty-one EEG electrodes were placed on the scalp, according to the International 10–20 system (Software: BrainRT, OSG Inc., Rumst, Belgium). A clinical neurophysiologist (NG), blinded to patients’ clinical features, reviewed all EEG recordings according to the definitions of the American Clinical Neurophysiology Society’s Standardized Critical Care EEG Terminology [17]. These EEG were subsequently classified into four categories based on background: mild/moderate encephalopathy (i.e., defined by the presence of reactivity), severe encephalopathy (i.e., defined by the absence of reactivity), burst-suppression (BS) and suppressed background (Additional file 1 for detail).

Reactivity was tested at least once daily by trained EEG technicians using a stimulation protocol comprising auditory, tactile and nociceptive stimulus. The presence of reactivity was assessed by the clinical neurophysiologists and defined as a clear and reproducible change in background amplitude or frequency, including attenuation of activity. Stimulus-induced period, rhythmic or ictal discharges (SIRPIDs) were not considered as the presence of reactivity if they were the only form of reactivity observed in the recording. No effort was made to decrease sedation prior to reactivity testing. Asymmetry was defined as the presence of a consistent asymmetry in amplitude between hemispheres or of a consistent asymmetry in frequency of ≥ 0.5 Hz, present for the majority (≥ 50%) of the recording. In addition, the occurrence of seizures and periodic discharges was also recorded.

### Outcome of the study

The primary outcome of this study was to report the occurrence of EEG abnormalities and assess their association with UO. Secondary outcome included the occurrence of EEG abnormalities in survivors versus non-survivors, in V–A versus V–V ECMO patients and in patients with cardiac arrest (CA) versus those without CA.

### Statistical analysis

Discrete variables were expressed as count (percentage) and continuous variables as mean ± standard deviation (SD) or median [25th–75th percentiles]. The Kolmogorov–Smirnov test was used, and histograms and normal-quantile plots were examined to verify the normality of distribution of continuous variables. Demographics, clinical and EEG patterns differences between groups (FO vs UO; survivors vs. non-survivors; V–V vs. V–A ECMO; CA vs. non-CA patients) were assessed using the chi-square test, Fisher’s exact test, Student’s t-test or Mann–Whitney U-test, as appropriate.

Multivariable logistic regression analysis with UO as the dependent variable was performed; collinearity between variables (i.e., a linear correlation coefficient higher than 0.3) was excluded prior to modeling; only variables associated with UO in the univariate analysis (*p* < 0.1) and the presence of cardiac arrest for clinical relevance were included in the multivariate model. We did not add in the model the variable “brain death” as it is part of the definition of death itself and was considered redundant with regard to study outcomes. Odds ratios (OR) with 95% confidence intervals (CI) were computed using an enter model. A similar approach was used to perform the multivariate analysis with hospital mortality as the dependent variable. We tested the fitness of the model using Hosmer and Lemeshow goodness-of-fit test. In case of no patients in the control group, we added in such group one patient to calculate OR, as differently it was not possible to obtain the value. The ORs of EEG background category were calculated using mild/moderate encephalopathy as reference. All statistical tests were two-tailed, and a p value < 0.05 was considered as statistically significant. Data were analyzed using IBM SPSS Statistics for Macintosh 25 (Armonk, NY, USA) and GraphPad PRISM version 8.0 (San Diego, CA, USA).

## Results

### Study population

**Fig. 1:**
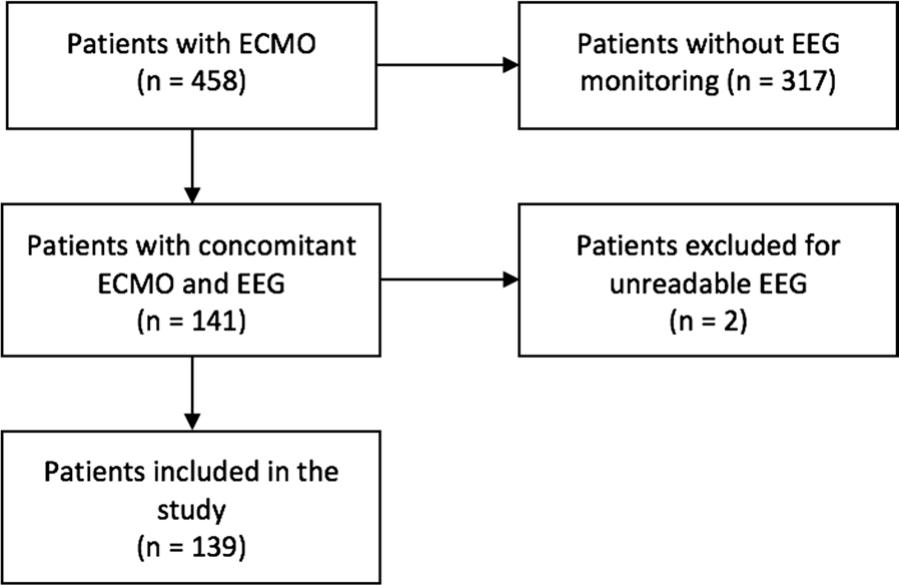
Flowchart of the study

Of 596 ECMO run in 458 patients over the study period (main characteristics in Additional file 2), 141 had concomitant EEG monitoring; 2 were excluded because EEG data were not interpretable, and 139 (median age 54 [41–62] years, 60 [43%] male gender) met the inclusion criteria and were included in the final analysis (Fig. 1). V–A ECMO was used in 98 (71%) of patients to treat either CA (*n* = 74) or cardiogenic shock (n = 24); V–V ECMO was used in 41 (29%) patients to treat acute respiratory distress syndrome (*n* = 33), as a bridge to transplant in decompensated end-stage lung diseases (*n* = 6) or for severe pneumonia (*n* = 2). A total of 73 cerebral CT-scan and 4 cerebral magnetic resonance imaging (MRI) were performed during the ICU stay; ischemic stroke or ICH were observed in 26 patients. The overall ICU length of stay was 9 [3–23] days; hospital mortality occurred in 91 (65%) patients and UO at 3 months was observed in 99 (71%) patients. Patients’ characteristics are summarized in Table 1 and Additional file 3.

**Table 1:** Characteristics of the study population, according to neurological outcome at 3 months (complete data are presented in Additional file 2: Additional Table 1)

	ALL (*n* = 139)	UO (*n* = 99)	FO (*n* = 40)	*p* value
Age (years)	54 [41–62]	56 [44–65]	48 [37–60]	0.06
Male gender, *n* (%)	60 (43)	45 (45)	15 (37)	0.45
Continuous EEG, *n* (%)	113 (81)	84 (85)	29 (72)	0.10
Cardiac arrest, *n* (%)	86 (62)	63 (64)	23 (57)	0.56
V–A ECMO, *n* (%)	98 (71)	74 (75)	24 (60)	0.10
Year of ECMO, *n* (%)				0.16
2009–2012	34 (25)	20 (20)	14 (35)	
2013–2015	50 (36)	39 (40)	11 (28)	
2016–2018	55 (39)	40 (40)	15 (37)	
Comorbidities				
COPD/Asthma, *n* (%)	18 (13)	13 (13)	5 (12)	1.00
Chronic hemodialysis, *n* (%)	18 (13)	14 (14)	4 (10)	0.59
Cirrhosis, *n* (%)	6 (4)	5 (5)	1 (2)	0.67
Heart failure (NYHA III-IV), *n* (%)	33 (24)	26 (26)	6 (15)	0.19
Immunosuppression, *n* (%)	22 (16)	15 (15)	7 (17)	0.80
Cancer, *n* (%)	7 (5)	9 (7)	–	0.19
Therapies				
Sedative drugs, *n* (%)	132 (95)	94 (95)	38 (95)	1.00
Analgesic drugs, *n* (%)	137 (99)	97 (98)	40 (100)	1.00
Antiepileptic drugs, *n* (%)	21 (15)	12 (12)	9 (22)	0.19
Complications				
Stroke/ICH, *n* (%)	26 (19)	23 (23)	3 (7)	0.03
Brain death, *n* (%)	15 (11)	15 (15)	–	0.01
Systemic bleeding, *n* (%)	33 (24)	26 (26)	7 (17)	0.38
Outcome variables				
ICU stay, days	9 [3–23]	7 [2–12]	26 [11–31]	< 0.01
Hospital stay, days	12 [3–51]	7 [2–15]	59 [40–86]	< 0.01
ICU death, *n* (%)	90 (65)	90 (91)	–	< 0.01
Hospital death, *n* (%)	91 (65)	91 (92)	–	< 0.01
GOS at 3 months	1 [1–4]	1 [1–1]	5 [5–5]	< 0.01
EEG findings				
Seizures /SE	11 (8)	9 (9)	2 (5)	0.51
GPDs/LPDs	10 (7)	8 (8)	2 (5)	0.72
Asymmetry	27 (19)	19 (19)	8 (20)	1.00
Background categories				< 0.001
Mild/moderate encephalopathy	87 (62)	52 (53)	35 (87)	
Severe encephalopathy	29 (21)	24 (24)	5 (13)	
Burst-suppression	4 (3)	4 (4)	0 (0)	
Suppressed background	19 (14)	19 (19)	0 (0)	

*UO* unfavorable outcome, *FO* favorable outcome, *EEG* electroencephalography, *V-A ECMO* veno-arterial extracorporeal membrane oxygenation, *COPD* chronic obstructive pulmonary disease, *NYHA* New York Heart Association, *ICH* intracranial hemorrhage, *ICU* Intensive Care Unit, *GOS* Glasgow Outcome Scale, *SE* status epilepticus, *GPDs* generalized periodic discharges, *LPDs* lateralized periodic discharges

### EEG monitoring

Continuous EEG was performed in 113 (81%) patients. The analysis of EEG background showed that 29 (21%) patients had severe encephalopathy, 4 (3%) had BS, and 19 (14%) a suppressed background. In addition, 11 (8%) of patients had seizures or status epilepticus, 10 (7%) had generalized periodic discharges (GPDs) or lateralized periodic discharges (LPDs), and 27 (19%) had asymmetry on EEG (Table 1). The median time from ECMO placement to EEG recording was 1 [0–2] days.

### EEG findings, neurological outcome and mortality

**Fig. 2:**
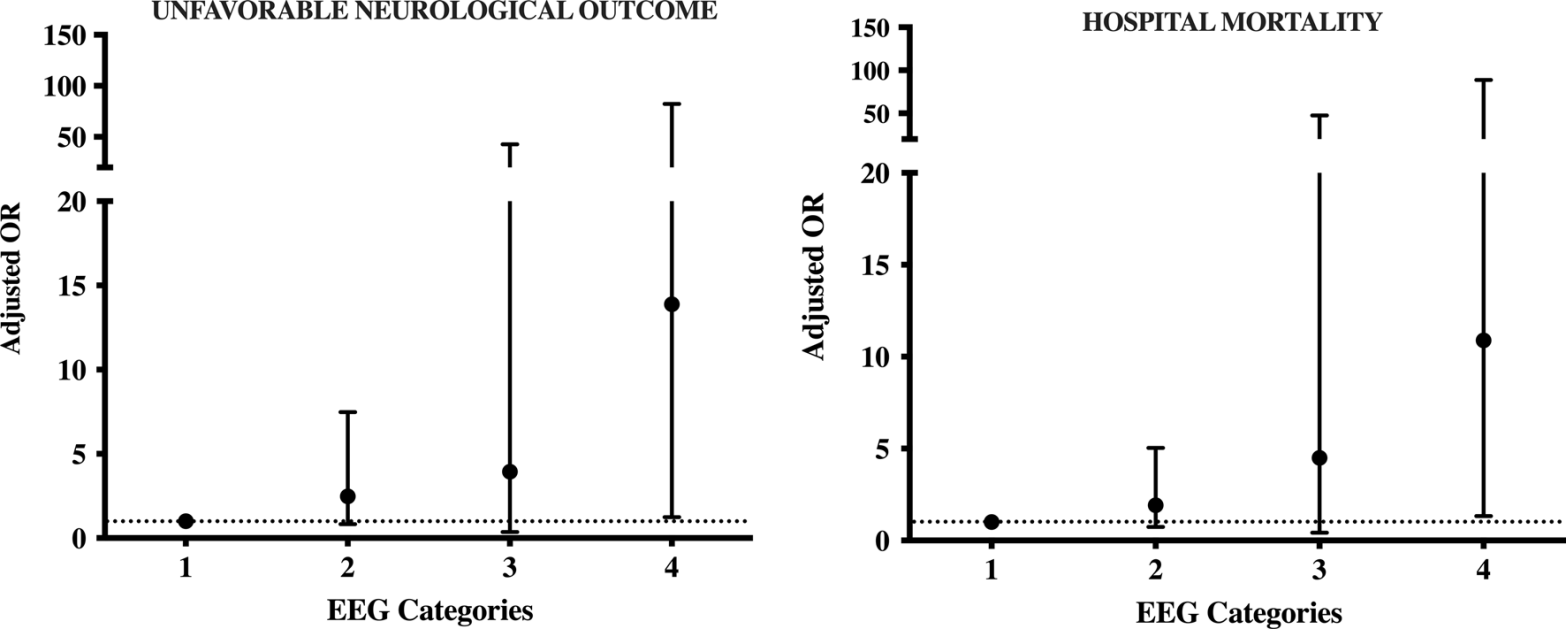
Adjusted odds ratio for unfavorable neurological outcome at 3 months and hospital mortality for the different categories of EEG background, using mild/moderate encephalopathy as reference (= 1); 2 = severe encephalopathy; 3 = burst-suppression; 4 = suppressed background

Patients with UO presented more frequently neurological complications (ischemic stroke, ICH or brain death) and had a higher ECMO blood flow, lactate levels as well as a lowest worst GCS during ECMO than those with FO (Table 1 and Additional file 3). On EEG analysis, patients with UO had more frequently a suppressed background than patients with FO (Table 1 and Additional file 3). In the multivariate analysis, after an adjustment for covariates (age, presence of cardiac arrest and high lactate levels) and considering all the EEG backgrounds, we observed that the occurrence of ischemic stroke or ICH (OR 4.57 [1.25–16.74]; *p* = 0.02) and suppressed background (OR 10.08 [1.24–82.20]; *p* = 0.03) were independently associated with UO (Fig. 2 and Table 2).

**Table 2:** Univariate and multivariate analysis to unfavorable neurological outcome at 3 months

	Univariate		Multivariate	
	Unadjusted OR [CI 95%]	*p* value	Adjusted OR [CI 95%]	*p* value
Age	1.02 [0.99–1.04]	0.10	1.02 [1.00–1.05]	0.10
Cardiac arrest	1.44 [0.70–3.00]	0.32	1.20 [0.52–2.73]	0.67
Lactate	1.11 [1.02–1.21]	0.02	1.08 [0.98–1.19]	0.14
Stroke/ICH	3.93 [1.11–13.91]	0.03	4.57 [1.25–16.74]	0.02
Background categories				
Mild/moderate encephalopathy	1		1	
Severe encephalopathy	3.23 [1.13–9.27]	0.03	2.48 [0.82–7.48]	0.11
Burst-suppression	2.69 [0.29–25.10]	0.39	3.94 [0.36–42.75]	0.26
Suppressed background	12.79 [1.64–99.94]	0.02	10.08 [1.24–82.20]	0.03

Hosmer and Lemeshow goodness-of-fit test: *p* = 0.04

Performing a secondary multivariate analysis and dichotomizing EEG according to reactivity, we observed that stroke or ICH (OR 3.85 [1.05–14.15]; *p* = 0.04) and presence of unreactive EEG (OR 5.39 [1.86–15.62]; *p* < 0.01) were independently associated with UO (Additional file 4). Equally, reactive EEG (OR 5.39 [1.86–15.62]; *p* < 0.01) and the absence of ischemic stroke or ICH (OR 3.85 [1.05–14.15]; *p* = 0.04) were independent predictors of favorable outcome (Additional file 5).

Non-survivors (15/91 had a diagnosis of brain death) had a higher ECMO blood flow and lactate levels than survivors. On EEG analysis, non-survivors had also more frequently a suppressed EEG background than survivors (Additional file 6). In the multivariate analysis, high lactate (OR 1.13 [1.02–1.24]; *p* = 0.02) and suppressed background (OR 10.88 [1.33–88.67]; *p* = 0.03) were independently associated with hospital mortality (Additional file 7).

### EEG findings and subgroup analyses

Patients on V–A ECMO had more frequently chronic heart failure and CA than those on V–V ECMO; also, lower gas flow, PaCO_2_, venous oxygen saturation and body temperature as well as higher lactate and glucose levels were observed in V–A than in V–V ECMO patients (Additional file 8). Despite V–A ECMO patients experienced more frequently brain death and major bleeding, ICU and hospital mortality was not significantly different than V–V ECMO patients. On EEG analysis, no differences were observed between the two groups.

Patients on ECMO after CA were more frequently on V–A configuration and on continuous EEG monitoring than others (Additional file 9); also, lower pH and body temperature as well as higher lactate and glucose levels were observed in CA patients when compared to the others. Despite the higher rate of brain death in CA patients, ICU and hospital mortality was not significantly different from non-CA patients. On EEG analysis, suppressed background was more frequently observed in CA patients compared to others (16 [19%] vs. 3 [6%]). Examples of one ECMO patient with EEG asymmetry associated with an ischemic stroke and another patient with seizures (before and after treatment) are shown in Additional files 10 and 11.

## Discussion

In this study evaluating EEG findings in a large cohort of adult ECMO patients, we observed that 38% of patients presented a severe EEG background abnormality (i.e., severe encephalopathy, BS or suppressed background) and 15% seizures or periodic discharges. The presence of suppressed EEG background was significantly associated with both UO and hospital mortality. The use of an EEG classification based on background analysis could be helpful to assess prognosis of adult ECMO patients, in particular for suppressed background. To the best of our knowledge, this is the first study reporting the independent prognostic role of EEG in a large ECMO population.

The literature is sparse on the use of EEG monitoring in adult patients on ECMO. In one study, 2 of the 13 adult patients had seizures and required specific antiepileptic medications; all EEGs had a background of predominant theta and delta frequencies and severe encephalopathy (i.e., poor variability and absence of reactivity) was associated with unfavorable neurologic outcome [8]. In another study including 25 adult patients on V-A ECMO, 95% of patients had a diffuse EEG slowing and 59% had discontinuous or an unreactive background; more severe findings were more frequently observed in patients with poor neurological outcome [14]. In a larger prospective cohort of V–A ECMO patients, low background frequency and an unreactive EEG were independently associated with poor outcome at 28 days [18]. Very low Bispectral Index (BIS) values, which is derived by a two-channel frontal EEG and suggest suppressed background or BS, were observed in adult patients undergoing urgent V–A ECMO cannulation for cardiac arrest and subsequently developing brain death [19]. In patients undergoing V–A ECMO for refractory cardiac arrest, malignant EEG patterns occurring within 96 h from admission were independently associated with poor neurological outcomes [20]. Our findings add important information on the role of EEG monitoring in this setting; although suppressed background was mainly observed in CA patients, this pattern may appear also in other ECMO patients and might be useful to perform further investigation to explain its etiology (i.e., diffuse injury, intracranial lesions and sedation). A further interesting and clinically relevant finding was related to the presence of epilepsy or status epilepticus (8% in our cohort) that in absence of EEG could not be detected and eventually treated. Moreover, severe EEG abnormalities were independently associated with UO and this was not related only to cerebrovascular complications, which are usually reported in ECMO patients [21]. Finally, EEG abnormalities were observed in both V–A and V–V configurations, suggesting a wide applicability of such monitoring in all ECMO patients.

Severe encephalopathy, BS and suppressed background have already been reported as reliable predictors of UO in different subgroups of critically ill patients. Azabou et al. showed that the absence of EEG reactivity (i.e., severe encephalopathy) was independently associated with the occurrence of delirium and ICU mortality in septic patients [11]. In comatose patients admitted for a hypoxic ischemic brain injury following CA, the presence of highly malignant EEG patterns (i.e., persistent suppressed background, BS or severe encephalopathy) was associated with a poor neurological outcome, with a high positive predictive value (94–97%) [13]. In mechanically ventilated patients, even short time in BS was independently associated with the occurrence of post-coma delirium and a delayed resolution of delirium, even after adjustment for covariates [22]. In both patients with traumatic brain injury or subarachnoid hemorrhage, neurological outcome was poor in most of the patients with absent EEG reactivity [23, 24]. Importantly, considering the requirement for a dedicated neurophysiologist and available devices, as well as the complexity of EEG interpretation, we advise caution on the wide use of EEG to prognosticate adult ECMO patient outcome until larger studies would validate this association with UO.

Our population showed a high rate of hospital mortality and poor neurological outcome; hence, comparison with previous studies remains difficult. Indeed, we enrolled many patients treated either with V–V or V–A ECMO that presented different diagnosis on ICU admission, as EEG monitoring has been implemented as neuromonitoring tool of critically ill patients in clinical practice. In previous studies, the rate of poor outcome ranged from 10 to 60%, as EEG was used in much selected cases, when a neurological complication was suspected. As neurological examination in ECMO patients is still limited by the concomitant use of sedatives, as these patients usually require sedation, EEG could be considered as a valuable tool to obtain relevant information about residual brain function, the occurrence of EEG abnormalities and changes in EEG background over time. Interestingly, the incidence of epileptiform EEG patterns in adult ECMO patients was quite limited and not associated with poor outcome, as antiepileptic drugs were rapidly initiated to limit these events.

This study presents some limitations. First, its retrospective design and subgroups analysis could provide important biases to the data interpretation. Second, we did not standardize the timing to initiated EEG monitoring in all our patients (except for patients suffering from CA who were monitored since day 1); as a consequence, we could have not missed early EEG abnormalities in some patients, which were potentially associated with poor outcome. Third, we used continuous EEG monitoring only in 81% of the patients; as a consequence, the incidence of electrographic seizures in patients with non-continuous EEG monitoring could have been underestimated. Fourth, we did not specifically analyze the morphology of BS, which may have influence the prognostic value of EEG, at least in CA patients [25]. Fifth, the role of sedation on EEG abnormalities still remains an open question; we did not specifically report cumulative sedative drugs, although the effects of sedation on EEG background are also dependent on other factors [26]. Moreover, patients on V–A ECMO patients were more frequently hypothermic during the first 24 h (i.e., temperature management after CA) and most of V–V ECMO patients deeply sedated in the early phase of therapy. Prospective studies evaluating the changes in EEG background due to sedation withdrawal in ECMO patients would be useful to quantify the impact of the sedative drugs on EEG abnormalities. Sixth, we did not specifically report reasons for death (e.g., withdrawal of life sustaining therapies, WLST), as these may impact on the prognostic role of monitoring tools. Similarly, it remains difficult to evaluate whether the EEG had contributed to decisions of WLST decisions, modifications of specific therapies or simply reflected underlying severe brain injury. Seventh, we did not compare EEG patterns with brain imaging or biomarkers of brain injury and this analysis might have been interesting to assess the correlation between anatomical and functional brain dysfunction in such patients. Finally, we did not observe a prognostic role for BS; however, only few patients presented this EEG abnormality.

## Conclusions

The use of EEG monitoring can be a useful tool to assess brain dysfunction and prognosis of patients undergoing ECMO support. Future studies will help to validate such findings and better describe EEG abnormalities of interest in this setting.

## Supplementary information

**Additional file 1** ECMO management and EEG definitions.

**Additional file 2** Main characteristics of ECMO patients, according to EEG monitoring.

**Additional file 3** Characteristics of the study population, according to neurological outcome at 3 months.

**Additional file 4** Univariate and multivariate analyses to predict unfavorable neurological outcome at 3 months, according to EEG reactivity.

**Additional file 5** Univariate and multivariate analyses to predict favorable neurological outcome at 3 months, according to EEG reactivity.

**Additional file 6** Characteristics of the study population, according to hospital mortality.

**Additional file 7** Univariate and Multivariate analysis to hospital mortality.

**Additional file 8** Characteristics of the study population, according to the ECMO configuration.

**Additional file 9** Characteristics of the study population, according to the occurrence of cardiac arrest.

**Additional file 10** Description of a case of early diagnose of stroke.

**Additional file 11** Description of a case of Nonconvulsive Status Epilepticus treated with success.

## Data Availability

The datasets used and/or analyzed during the current study are available from the corresponding author on reasonable request.

## Abbreviations

ECMO: Extracorporeal membrane oxygenation
EEG: Electroencephalography
NIRS: Near-infrared spectroscopy
V–A: Veno–arterial
V–V: Veno–venous
ICU: Intensive Care Unit
FiO_2_: Fraction of inspired oxygen
PaO_2_: Partial pressure of oxygen in arterial blood
PaCO_2_: Partial pressure of oxygen in arterial blood
ICH: Intracranial hemorrhage
RBC: Red blood cells
RBCT: Red blood cells transfusion
GOS: Glasgow outcome scale
FO: Favorable outcome
UO: Unfavorable outcome
BS: Burst-suppression
CA: Cardiac arrest
SD: Standard deviation
OR: Odds ratio
CI: Confidence interval
