# Supplementary material for: Electroencephalographic features in patients undergoing extracorporeal membrane oxygenation
Source: Crit Care. 2020 Oct 30;24:629. doi: 10.1186/s13054-020-03353-z (PMC7598240; doi:10.1186/s13054-020-03353-z)
Supplement: Supplementary file 1 — Additional file 1 ECMO management and EEG definitions. [file 13054_2020_3353_MOESM1_ESM.docx]

**Additional File 1**

**Methods**

*ECMO management*

V-A ECMO was used for the patients admitted after in- or out-of-hospital cardiac arrest (CA) or for cardiogenic shock due to different acute causes (i.e. ischemic cardiomyopathy, myocarditis, septic cardiomyopathy, severe and refractory arrhythmias, toxics, post-transplantation left or right ventricular failure). Veno-venous (V-V) ECMO was used to treat patients with severe respiratory failure who failed common ventilator and medical therapy. All ECMO equipment was implanted with peripheral (the most frequent configuration was femoro-femoral in V-A and femoro-jugular if V-V) heparin-coated cannulation (18–22 Fr arterial cannula and 21–25 Fr venous cannula; Edwards Lifesciences, Irvine, CA). A centrifugal blood pump (RevolutionTM blood pump; Sorin, Milan, Italy) was initially set at a blood flow of 3-5 L/min (based on patient body surface area), according to clinical needs. Priming of the ECMO circuit consisted of 700 mL of Plasmalyte solution (Baxter Healthcare Corp., Deerfield, IL). With peripheral V-A implantation, an anterograde single-lumen 8 Fr catheter (Arrow Inc., Reading, PA) was placed to avoid limb ischaemia. A heat exchanger (Blanketrol II; Sub-Zero Products Inc., Cincinnati, OH) was used to maintain body temperature at 37°C, when indicated. Systemic anticoagulation was achieved by intravenous administration of unfractionated heparin. In V-A ECMO patients developing differential hypoxia despite increasing ECMO blood flows, different interventions, including reducing inotropes, optimizing mechanical ventilation or giving diuretics, were initiated and the conversion to hybrid configuration (i.e. a third venous reinjection cannula) was considered.

*EEG definitions*

Definitions of EEG patterns were as follows:

1) Mild/moderate encephalopathy was defined as an excess of slow activity (theta and delta frequencies) and presence of spontaneous variability and/or reactivity of the EEG to external stimulation.

2) Severe encephalopathy was defined as either a monotonous slow and/or attenuated activity with absence of reactivity and variability or a discontinuous EEG background, characterized by the presence of periods of diffuse attenuation lasting <50 % of the EEG epoch.

3) Suppression-Burst (SB) pattern was defined by the presence of bursts of polyrhythmic activity alternating with periods of suppression (suppression EEG) lasting more than 50 % of the epoch.

4) Suppression was defined by the absence of any EEG activity (<10 μV) during the entire epoch.

5) Seizures were defined as generalized spike-wave discharges at 3/s or faster or clearly evolving discharges of any type that reached a frequency >4/s, whether focal or generalized). Status epilepticus (SE) was defined as a single epileptic seizure lasting more than five minutes or two or more seizures within a five-minute period with an incomplete return to consciousness. Previous definitions used a 30-minute time limit. The seizures could either be of the tonic-clonic type with a regular pattern of contraction and extension of the arms and legs or of types that do not involve contractions (non-convulsive status epilepticus, NCSE).

6) Periodic discharges (lateralized and generalized: LPDs and GPDs, respectively) were defined as repetition of high amplitude theta or delta waves, sharp or not, occurring repeatedly, generally anteriorly predominant, with a regular frequency, and morphology. We also searched for sporadic epileptiform discharges (SEDs).
