# Supplementary material for: Electroencephalographic features in patients undergoing extracorporeal membrane oxygenation
Source: Crit Care. 2020 Oct 30;24:629. doi: 10.1186/s13054-020-03353-z (PMC7598240; doi:10.1186/s13054-020-03353-z)
Supplement: Supplementary file 2 — Additional file 2 Main characteristics of ECMO patients, according to EEG monitoring. [file 13054_2020_3353_MOESM2_ESM.docx]

**Additional File 2**

**Supplemental Table 1.** Main characteristics of ECMO patients, according to EEG monitoring

|  | **EEG MONITORING (n = 139)** | **NO-EEG MONITORING**  **(n = 317)** | ***p values*** |
| --- | --- | --- | --- |
| Age | 54 [41-62] | 53 [40-62] | 0.57 |
| Cardiac Arrest | 86 (62) | 87 (28) | <0.01 |
| V-A ECMO | 98 (71) | 177 (55) | <0.01 |
| Hospital Mortality | 91 (65) | 214 (67) | 0.67 |
| *V-A ECMO* | 69 (50) | 141 (44) | 0.10 |
| *V-V ECMO* | 22 (15) | 73 (23) | 0.99 |

V-A ECMO = Veno-arterial Extracorporeal Membrane Oxygenation; V-V ECMO= Veno-venous Extracorporeal Membrane Oxygenation
