# Supplementary material for: Electroencephalographic features in patients undergoing extracorporeal membrane oxygenation
Source: Crit Care. 2020 Oct 30;24:629. doi: 10.1186/s13054-020-03353-z (PMC7598240; doi:10.1186/s13054-020-03353-z)
Supplement: Supplementary file 3 — Additional file 3 Characteristics of the study population, according to neurological outcome at 3 months. [file 13054_2020_3353_MOESM3_ESM.docx]

**Additional File 3**

**Supplemental Table 2.** Characteristics of the study population, according to neurological outcome at 3 months.

|  | **ALL**  **(n=139)** | **UO**  **(n=99)** | **FO**  **(n=40)** | ***p value*** |
| --- | --- | --- | --- | --- |
| Age, (years) | 54 [41-62] | 56 [44-65] | 48 [37-60] | 0.06 |
| Male Gender, n (%) | 60 (43) | 45 (45) | 15 (37) | 0.45 |
| Continuous EEG, n (%) | 113 (81) | 84 (85) | 29 (72) | 0.10 |
| Cardiac arrest, n (%) | 86 (62) | 63 (64) | 23 (57) | 0.56 |
| Year of ECMO, n (%)  2009-2012  2013-2015  2016-2018 | 34 (25)  50 (36)  55 (39) | 20 (20)  39 (40)  40 (40) | 14 (35)  11 (28)  15 (37) | 0.16 |
| COPD/Asthma, n (%) | 18 (13) | 13 (13) | 5 (12) | 1.00 |
| Chronic Hemodialysis, n (%) | 18 (13) | 14 (14) | 4 (10) | 0.59 |
| Cirrhosis, n (%) | 6 (4) | 5 (5) | 1 (2) | 0.67 |
| Heart failure (NYHA III-IV), n (%) | 33 (24) | 26 (26) | 6 (15) | 0.19 |
| Immunosuppression, n (%) | 22 (16) | 15 (15) | 7 (17) | 0.80 |
| Cancer, n (%) | 7 (5) | 9 (7) | - | 0.19 |
|  |  |  |  |  |
| **ECMO Management** |  |  |  |  |
| ECMO V-A, n (%) | 98 (71) | 74 (75) | 24 (60) | 0.10 |
| Blood flow, L/min | 4.0 [3.5-4.6] | 4.0 [3.6-4.7] | 3.7 [3.3-4.2] | 0.02 |
| Gas flow, L/min | 5.0 [3.0-6.0] | 4.5 [3.0-6.0] | 4.0 [3.0-6.0] | 0.73 |
| Anticoagulation, n (%) | 100 (72) | 68 (69) | 32 (80) | 0.21 |
| RBC transfusion, n (%) | 89 (64) | 65 (66) | 24 (60) | 0.56 |
|  |  |  |  |  |
| **Clinical variables and therapies** |  |  |  |  |
| Lowest pH | 7.28 [7.18-7.35] | 7.27 [7.16-7.35] | 7.30 [7.19-7.35] | 0.30 |
| Lowest PaCO_2_, mmHg | 32 [28-34] | 32 [27-34] | 31 [28-35] | 0.98 |
| Lowest PaO_2_, mmHg | 64 [58-72] | 63 [58-70] | 66 [61-72] | 0.37 |
| Lowest Hb, g/dl | 7.4 [6.8-8.4] | 7.3 [6.9-8.3] | 7.5 [6.7-8.1] | 0.44 |
| Lowest MAP, mmHg | 63 [59-67] | 63 [59-67] | 65 [60-67] | 0.20 |
| Lowest ScvO_2_, % | 66 [59-76] | 66 [59-76] | 67 [59-76] | 0.94 |
| Highest Lactate, mmol/L | 5 [2.6-9.4] | 6.0 [3.3-10.6] | 4.3 [2.3-8.1] | 0.05 |
| Lowest temperature, °C | 35.0 [33.5-36.0] | 34.8 [33.5-35.9] | 35.4 [33.2-36.2] | 0.66 |
| Highest glycemia, mg/dL | 196 [165-297] | 205 [166-317] | 181 [163-230] | 0.30 |
| Lowest glycemia, mg/dL | 89 [74-108] | 89 [73-109] | 85 [74-99] | 0.44 |
| Worst GCS during ECMO | 3 [3-3] | 3 [3-3] | 3 [3-3] | 0.04 |
| Sedative drugs, n (%) | 132 (95) | 94 (95) | 38 (95) | 1.00 |
| Analgesic drugs, n (%) | 137 (99) | 97 (98) | 40 (100) | 1.00 |
| Antiepileptic drugs, n (%) | 21 (15) | 12 (12)# | 9 (22)# | 0.19 |
| Leviracetam, n (%) | 21 (15) | 12 (12)# | 9 (22)# | 0.19 |
| Valproate, n (%) | 7 (5) | 5 (5) | 2 (5) | 1 |
|  |  |  |  |  |
| **Complications** |  |  |  |  |
| Stroke/ICH, n (%) | 26 (19) | 23 (23) | 3 (7) | 0.03 |
| Brain death, n (%) | 15 (11) | 15 (15) | - | 0.01 |
| Systemic Bleeding, n (%) | 33 (24) | 26 (26) | 7 (17) | 0.38 |
|  |  |  |  |  |
| **Outcome Variables** |  |  |  |  |
| ICU stay, days | 9 [3-23] | 7 [2-12] | 26 [11-31] | <0.01 |
| Hospital stay, days | 12 [3-51] | 7 [2-15] | 59 [40-86] | <0.01 |
| ICU death, n (%) | 90 (65) | 90 (91) | - | <0.01 |
| Hospital death, n (%) | 91 (65) | 91 (92) | - | <0.01 |
| GOS at 3 months | 1 [1-4] | 1 [1-1] | 5 [5-5] | <0.01 |
|  |  |  |  |  |
| **EEG Findings** |  |  |  |  |
| Seizures /SE | 11 (8) | 9 (9) | 2 (5) | 0.51 |
| GPDs/LPDs | 10 (7) | 8 (8) | 2 (5) | 0.72 |
| Asymmetry | 27 (19) | 19 (19) | 8 (20) | 1.00 |
| Background Categories  *Mild/Moderate Encephalopathy*  *Severe Encephalopathy*  *Burst-Suppression*  *Suppressed Background* | 87 (62)  29 (21)  4 (3) 19 (14) | 52 (53)  24 (24)  4 (4) 19 (19) | 35 (87)  5 (13) 0 (0) 0 (0) | < 0.001 |

EEG= Electroencephalography; COPD= Chronic Obstructive Pulmonary Disease; NYHA= New York Heart Association; V-A ECMO = Veno-arterial Extracorporeal Membrane Oxygenation; V-V ECMO= Veno-venous Extracorporeal Membrane Oxygenation; RBC = Red Blood Cells; MAP = Mean Arterial Pressure; GCS = Glasgow Coma Scale; ICH = Intracranial Hemorrhage; ICU = Intensive Care Unit; GOS = Glasgow Outcome Scale; SE = Status Epilepticus; GPDs = Generalized Periodic Discharges; LPDs = Lateralized Periodic Discharges.
