# Supplementary material for: Electroencephalographic features in patients undergoing extracorporeal membrane oxygenation
Source: Crit Care. 2020 Oct 30;24:629. doi: 10.1186/s13054-020-03353-z (PMC7598240; doi:10.1186/s13054-020-03353-z)
Supplement: Supplementary file 4 — Additional file 4 Univariate and multivariate analyses to predict unfavorable neurological outcome at 3 months, according to EEG reactivity. [file 13054_2020_3353_MOESM4_ESM.docx]

**Additional File 4**

**Supplemental Table 3:** Univariate and multivariate analyses to predict unfavorable neurological outcome at 3 months, according to reactivity.

|  | **UNIVARIATE** | | **MULTIVARIATE** | |
| --- | --- | --- | --- | --- |
|  | **Unadjusted OR [CI 95%]** | ***p value*** | **Adjusted OR [CI 95%]** | ***p value*** |
| Age | 1.02 [1.00 – 1.04] | 0.09 | 1.02 [1.00 – 1.05] | 0.07 |
| Cardiac Arrest | 1.29 [0.61 – 2.74] | 0.50 | 1.08 [0.46 – 2.51] | 0.87 |
| Lactate | 1.10 [1.01 – 1.21] | 0.03 | 1.07 [0.97 – 1.17] | 0.20 |
| Stroke/ICH | 3.73 [1.05 – 13.23] | 0.04 | 3.85 [1.05 – 14.15] | 0.04 |
| Unreactive EEG | 6.33 [2.29 – 17.49] | <0.01 | 5.39 [1.86 – 15.62] | <0.01 |

*Hosmer and Lemeshow goodness-of-fit test: p=0.01*
