# Supplementary material for: Electroencephalographic features in patients undergoing extracorporeal membrane oxygenation
Source: Crit Care. 2020 Oct 30;24:629. doi: 10.1186/s13054-020-03353-z (PMC7598240; doi:10.1186/s13054-020-03353-z)
Supplement: Supplementary file 5 — Additional file 5 Univariate and multivariate analyses to predict favorable neurological outcome at 3 months, according to EEG reactivity. [file 13054_2020_3353_MOESM5_ESM.docx]

**Additional File 5**

**Supplemental Table 4:** Univariate and multivariate analyses to predict favorable neurological outcome at 3 months.

|  | **UNIVARIATE** | | **MULTIVARIATE** | |
| --- | --- | --- | --- | --- |
|  | **Unadjusted OR [CI 95%]** | ***p value*** | **Adjusted OR [CI 95%]** | ***p value*** |
| Age | 0.98 [0.96 – 1.00] | 0.09 | 0.98 [0.95 – 1.00] | 0.07 |
| Cardiac Arrest | 0.77 [0.36 – 1.64] | 0.50 | 0.93 [0.40 – 2.17] | 0.87 |
| Lactate | 0.91 [0.83 – 0.99] | 0.03 | 0.94 [0.85 – 1.04] | 0.20 |
| Absence of Stroke/ICH | 3.73 [1.05 – 13.23] | 0.04 | 3.85 [1.05 – 14.15] | 0.04 |
| Reactive EEG | 6.33 [2.29 – 17.49] | <0.01 | 5.39 [1.86 – 15.62] | <0.01 |

*Hosmer and Lemeshow goodness-of-fit test: p=0.01*
