# Supplementary material for: Electroencephalographic features in patients undergoing extracorporeal membrane oxygenation
Source: Crit Care. 2020 Oct 30;24:629. doi: 10.1186/s13054-020-03353-z (PMC7598240; doi:10.1186/s13054-020-03353-z)
Supplement: Supplementary file 6 — Additional file 6 Characteristics of the study population, according to hospital mortality. [file 13054_2020_3353_MOESM6_ESM.docx]

**Additional File 6**

**Supplemental Table 5.** Characteristics of the study population, according to hospital mortality.

|  | NON-SURVIVORS  (n=91) | SURVIVORS  (n=48) | *p value* |
| --- | --- | --- | --- |
| Age, (years) | 56 [44-65] | 49 [37-60] | 0.09 |
| Male Gender, n (%) | 42 (46) | 18 (37) | 0.37 |
| Continuous EEG, n (%) | 76 (83) | 37 (77) | 0.37 |
| Cardiac arrest, n (%)  Year of ECMO, n (%) | 59 (65) | 27 (56) | 0.36  0.61 |
| 2009-2012  2013-2015  2016-2018 | 20 (22)  33 (36)  38 (42) | 14 (30)  17 (35)  17 (35) |  |
|  |  |  |  |
| *Comorbidities* |  |  |  |
| COPD/Asthma, n (%) | 11 (12) | 7 (15) | 0.79 |
| Chronic Hemodialysis, n (%) | 14 (15) | 4 (8) | 0.30 |
| Cirrhosis, n (%) | 5 (5) | 1 (2) | 0.66 |
| Heart failure (NYHA III-IV), n (%) | 23 (25) | 9 (19) | 0.52 |
| Immunosuppression, n (%) | 13 (14) | 9 (18) | 0.63 |
| Cancer, n (%) | 7 (8) | - | 0.10 |
|  |  |  |  |
| *ECMO Management* |  |  |  |
| ECMO VA, n (%) | 69 (76) | 29 (60) | 0.08 |
| Blood flow, L/min | 4.0 [3.7-4.7] | 3.7 [3.2-4.2] | 0.01 |
| Gas flow, L/min | 4.25 [3.0-6.0] | 4.0 [3-6] | 0.98 |
| Anticoagulation, n (%) | 62 (68) | 38 (79) | 0.23 |
| RBC transfusion, n (%) | 61 (67) | 28 (58) | 0.35 |
|  |  |  |  |
| *Clinical variables and therapies* |  |  |  |
| Lowest pH | 7.26 [7.13-7.34] | 7.30 [7.21-7.36] | 0.06 |
| Lowest PaCO_2_, mmHg | 32 [27-34] | 32 [28-35] | 0.85 |
| Lowest PaO_2_, mmHg | 63 [58-71] | 66 [59-71] | 0.62 |
| Lowest Hb, g/dl | 7.3 [6.8-8.3] | 7.6 [6.8-8.1] | 0.40 |
| Lowest MAP, mmHg | 62 [58-67] | 65 [62-66] | 0.22 |
| Lowest ScvO_2_, % | 65 [59-76] | 66 [59-76] | 0.99 |
| Highest Lactate, mmol/L | 6.5 [3.5-11.5] | 4.2 [2.2-7.9] | <0.01 |
| Lowest temperature, °C | 34.7 [33.5-35.9] | 35.2 [33.4-36.2] | 0.61 |
| Highest glycemia, mg/dL | 206 [171-317] | 181 [161-252] | 0.26 |
| Lowest glycemia, mg/dL | 89 [73-110] | 86 [74-99] | 0.22 |
| Worst GCS during ECMO | 3 [3-3] | 3 [3-3] | 0.13 |
| Sedative drugs, n (%) | 87 (96) | 45 (94) | 0.69 |
| Analgesic drugs, n (%) | 90 (99) | 47 (98) | 1.00 |
| Antiepileptic drugs, n (%) | 12 (13) | 9 (19) | 0.46 |
| Leviracetam, n (%) | 12 (13) | 9 (19) | 0.46 |
| Valproate, n (%) | 5 (5) | 2 (4) | 1.00 |
|  |  |  |  |
| *Complications* |  |  |  |
| Stroke/ICH, n (%) | 21 (23) | 5 (10) | 0.11 |
| Brain death, n (%) | 15 (16) | - | <0.01 |
| Bleeding, n (%) | 24 (26) | 9 (19) | 0.40 |
|  |  |  |  |
| *Outcome variables* |  |  |  |
| ICU stay, days | 5 [2-10] | 27 [14-33] | <0.01 |
| Hospital stay, days | 6.5 [2-12.5] | 63 [40-98] | <0.01 |
| ICU death, n (%) | 90 (99) | - | <0.01 |
| GOS at 3 months | 1 [1-1] | 5 [4-5] | <0.01 |
| Poor neurological outcome, n (%) | 91 (100) | 8 (17) | <0.01 |
|  |  |  |  |
| *EEG findings* |  |  |  |
| Seizures/SE, n (%) | 9 (10) | 2 (4) | 0.33 |
| GPDs/LPDs, n (%) | 8 (9)# | 2 (4)# | 0.49 |
| Asymmetry, n (%) | 19 (21) | 8 (17) | 0.66 |
| Background Categories  *Mild/Moderate Encephalopathy, n (%)*  *Severe Encephalopathy, n (%)* | 47 (52)  21 (23) | 40 (83)  8 (17) | < 0.01 |
| *Burst-Suppression, n (%)* | 4 (4) | 0 (0) |  |
| *Suppressed Background, n (%)* | 19 (21)* | 0 (0)* |  |

EEG= Electroencephalography; COPD= Chronic Obstructive Pulmonary Disease; NYHA= New York Heart Association; V-A ECMO = Veno-arterial Extracorporeal Membrane Oxygenation; V-V ECMO= Veno-venous Extracorporeal Membrane Oxygenation; RBC = Red Blood Cells; MAP = Mean Arterial Pressure; GCS = Glasgow Coma Scale; ICH = Intracranial Hemorrhage; ICU = Intensive Care Unit; GOS = Glasgow Outcome Scale; SE = Status Epilepticus; GPDs = Generalized Periodic Discharges; LPDs = Lateralized Periodic Discharges.
