# Supplementary material for: Electroencephalographic features in patients undergoing extracorporeal membrane oxygenation
Source: Crit Care. 2020 Oct 30;24:629. doi: 10.1186/s13054-020-03353-z (PMC7598240; doi:10.1186/s13054-020-03353-z)
Supplement: Supplementary file 7 — Additional file 7 Univariate and Multivariate analysis to hospital mortality. [file 13054_2020_3353_MOESM7_ESM.docx]

**Additional File 7**

**Supplemental Table 6.** Univariate and Multivariate analysis to hospital mortality.

|  | **UNIVARIATE** | | **MULTIVARIATE** | |
| --- | --- | --- | --- | --- |
|  | **Unadjusted OR [CI 95%]** | ***p value*** | **Adjusted OR [CI 95%]** | ***p value*** |
| Age | 1.02 [0.99 – 1.04] | 0.12 | 1.02 [0.99 – 1.04] | 0.10 |
| Cardiac Arrest | 1.57 [0.77 – 3.17] | 0.21 | 1.14 [0.52 – 2.48] | 0.74 |
| Lactate | 1.15 [1.06 – 1.27] | <0.01 | 1.13 [1.02 – 1.24] | 0.02 |
| Background Categories  *Mild/Moderate Encephalopathy*  *Severe Encephalopathy*  *Burst-Suppression*  *Suppressed Background* | 1  2.23 [0.89 – 5.59]  3.40 [0.37 – 31.70]  16.17 [2.07 – 126.19] | 0.09  0.28  <0.01 | 1  1.92 [0.74 – 5.04]  4.49 [0.42 – 47.44]  10.88 [1.33 – 88.67] | 0.18  0.21  0.03 |

*Hosmer and Lemeshow goodness-of-fit test: p=0.44*
