# Supplementary material for: Electroencephalographic features in patients undergoing extracorporeal membrane oxygenation
Source: Crit Care. 2020 Oct 30;24:629. doi: 10.1186/s13054-020-03353-z (PMC7598240; doi:10.1186/s13054-020-03353-z)
Supplement: Supplementary file 8 — Additional file 8 Characteristics of the study population, according to the ECMO configuration. [file 13054_2020_3353_MOESM8_ESM.docx]

**Additional File 8**

**Supplemental Table 7.** Characteristics of the study population, according to the ECMO configuration.

|  | V-A ECMO  (n=98) | V-V ECMO  (n=41) | *p value* |
| --- | --- | --- | --- |
| Age, (years) | 55 [44-63] | 48 [37-62] | 0.07 |
| Male Gender, n (%) | 41 (42) | 19 (46) | 0.71 |
| Continuous EEG, n (%) | 80 (82) | 33 (81) | 1.00 |
| Cardiac arrest, n (%) | 74 (76) | 12 (29) | <0.01 |
|  |  |  |  |
| *Comorbidities* |  |  |  |
| COPD/Asthma, n (%) | 10 (10) | 8 (20) | 0.17 |
| Chronic Hemodialysis, n (%) | 16 (16) | 2 (5) | 0.10 |
| Cirrhosis, n (%) | 4 (4) | 2 (5) | 1.00 |
| Heart failure (NYHA III-IV), n (%) | 28 (29) | 4 (10) | 0.02 |
| Immunosuppression, n (%) | 12 (12) | 10 (24) | 0.08 |
| Cancer, n (%) | 4 (4) | 3 (7) | 0.42 |
|  |  |  |  |
| *ECMO Management* |  |  |  |
| ECMO VA, n (%) | 98 (100) | - |  |
| Blood flow, L/min | 4 [3.5-4.5] | 4 [3.3-4.9] | 0.97 |
| Gas flow, L/min | 4 [3-5.5] | 5 [4-7] | 0.01 |
| Anticoagulation, n (%) | 67 (68) | 33 (81) | 0.21 |
| RBC transfusion, n (%) | 59 (60) | 30 (73) | 0.18 |
|  |  |  |  |
| *Clinical variables and therapies* |  |  |  |
| Lowest pH | 7.27 [7.16-7.35] | 7.30 [7.20-7.35] | 0.52 |
| Lowest PaCO_2_, mmHg | 31 [26-33] | 33 [30-36] | 0.01 |
| Lowest PaO_2_, mmHg | 65 [59-76] | 63 [57-68] | 0.16 |
| Lowest Hb, g/dl | 7.4 [6.9-8.2] | 7.2 [6.7-8.7] | 0.90 |
| Lowest MAP, mmHg | 63 [57-67] | 64 [61-67] | 0.63 |
| Lowest ScvO_2_, % | 64 [59-73] | 69 [63-85] | 0.01 |
| Highest Lactate, mmol/L | 5.7 [3.5-9.9] | 4 [1.9-8.6] | 0.02 |
| Lowest temperature, °C | 34.2 [33.2-35.7] | 35.9 [35.1-36.5] | <0.01 |
| Highest glycemia, mg/dL | 213 [174-333] | 174 [158-223] | <0.01 |
| Lowest glycemia, mg/dL | 87 [70-108] | 90 [80-104] | 0.88 |
| Worst GCS during ECMO | 3 [3-3] | 3 [3-3] | 0.83 |
| Sedative drugs, n (%) | 95 (97) | 37 (90) | 0.20 |
| Analgesic drugs, n (%) | 97 (99) | 40 (98) | 0.50 |
| Antiepileptic drugs, n (%) | 13 (13) | 8 (20) | 0.44 |
| Leviracetam, n (%) | 13 (13) | 8 (20) | 0.44 |
| Valproate, n (%) | 5 (5) | 2 (5) | 1.00 |
|  |  |  |  |
| *Complications* |  |  |  |
| Stroke/ICH, n (%) | 21 (21) | 5 (12) | 0.24 |
| Brain death, n (%) | 15 (15) | 0 (0) | 0.01 |
| Bleeding, n (%) | 28 (29) | 5 (12) | 0.02 |
|  |  |  |  |
| *Outcome variables* |  |  |  |
| ICU stay, days | 8 [2-20] | 15 [9-31] | 0.01 |
| Hospital stay, days | 10 [3-40] | 21 [9-65] | 0.04 |
| ICU death, n (%) | 68 (69) | 22 (54) | 0.08 |
| Hospital death, n (%) | 69 (70) | 22 (54) | 0.08 |
| GOS at 3 months | 1 [1-3] | 1 [1-5] | 0.08 |
| Poor neurological outcome, n (%) | 74 (76) | 25 (61) | 0.10 |
|  |  |  |  |
| *EEG findings* |  |  |  |
| Seizures/SE, n (%) | 6 (6) | 5 (12) | 0.30 |
| GPDs/LPDs, n (%) | 6 (6) | 4 (10) | 0.48 |
| Asymmetry, n (%) | 20 (20) | 9 (22) | 0.82 |
| Background Categories  *Mild/Moderate Encephalopathy, n (%)*  *Severe Encephalopathy, n (%)* | 60 (61)  20 (20) | 27 (66)  9 (22) | 0.84 |
| *Burst-Suppression, n (%)* | 3 (3) | 1 (2) |  |
| *Suppressed Background, n (%)* | 15 (15) | 4 (10) |  |

EEG= Electroencephalography; COPD= Chronic Obstructive Pulmonary Disease; NYHA= New York Heart Association; V-A ECMO = Veno-arterial Extracorporeal Membrane Oxygenation; V-V ECMO= Veno-venous Extracorporeal Membrane Oxygenation; RBC = Red Blood Cells; MAP = Mean Arterial Pressure; GCS = Glasgow Coma Scale; ICH = Intracranial Hemorrhage; ICU = Intensive Care Unit; GOS = Glasgow Outcome Scale; SE = Status Epilepticus; GPDs = Generalized Periodic Discharges; LPDs = Lateralized Periodic Discharges.
