# Supplementary material for: Electroencephalographic features in patients undergoing extracorporeal membrane oxygenation
Source: Crit Care. 2020 Oct 30;24:629. doi: 10.1186/s13054-020-03353-z (PMC7598240; doi:10.1186/s13054-020-03353-z)
Supplement: Supplementary file 9 — Additional file 9 Characteristics of the study population, according to the occurrence of cardiac arrest. [file 13054_2020_3353_MOESM9_ESM.docx]

**Additional File 9**

**Supplemental Table 8.** Characteristics of the study population, according to the occurrence of cardiac arrest.

|  | CARDIAC ARREST  (n=86) | NON CARDIAC ARREST  (n=53) | *p value* |
| --- | --- | --- | --- |
| Age, (years) | 54 [43-62] | 53 [41-65] | 0.72 |
| Male Gender, n (%) | 36 (42) | 24 (45) | 0.73 |
| Continuous EEG, n (%) | 76 (88) | 37 (70) | 0.01 |
|  |  |  |  |
| *Comorbidities* |  |  |  |
| COPD/Asthma, n (%) | 8 (9) | 10 (19) | 0.12 |
| Chronic Hemodialysis, n (%) | 10 (12) | 8 (15) | 0.61 |
| Cirrhosis, n (%) | 2 (2) | 4 (8) | 0.20 |
| Heart failure (NYHA III-IV), n (%) | 19 (22) | 13 (25) | 0.84 |
| Immunosuppression, n (%) | 8 (9) | 14 (26) | 0.01 |
| Cancer, n (%) | 4 (5) | 3 (6) | 1.00 |
|  |  |  |  |
| *ECMO Management* |  |  |  |
| ECMO VA, n (%) | 74 (86) | 24 (45) | 0.01 |
| Blood flow, L/min | 4 [3.5-4.5] | 4 [3.3-4.5] | 0.78 |
| Gas flow, L/min | 4 [3-6] | 4 [3-6] | 0.87 |
| Anticoagulation, n (%) | 59 (69) | 41 (77) | 0.33 |
| RBC transfusion, n (%) | 57 (66) | 32 (60) | 0.59 |
|  |  |  |  |
| *Clinical variables and therapies* |  |  |  |
| Lowest pH | 7.23[7.11-7.32] | 7.32 [7.23-7.37] | <0.01 |
| Lowest PaCO_2_, mmHg | 32 [26-34] | 32 [28-35] | 0.53 |
| Lowest PaO_2_, mmHg | 65 [60-72] | 63 [57-71] | 0.18 |
| Lowest Hb, g/dl | 7.3 [6.8-8.1] | 7.5 [7-8.8] | 0.42 |
| Lowest MAP, mmHg | 63 [58-67] | 64 [60-67] | 0.57 |
| Lowest ScvO_2_, % | 68 [59-76] | 65 [59-77] | 0.47 |
| Highest Lactate, mmol/L | 5.7 [3.6-11.1] | 4.2 [2-7.9] | <0.01 |
| Lowest temperature, °C | 34 [33.1-35] | 36.2 [35.6-36.7] | <0.01 |
| Highest glycemia, mg/dL | 212 [171-350] | 189 [159-241] | 0.03 |
| Lowest glycemia, mg/dL | 88 [73-104] | 86 [77-109] | 0.92 |
| Worst GCS during ECMO | 3 [3-3] | 3 [3-3] | 0.56 |
| Sedative drugs, n (%) | 83 (97) | 49 (93) | 0.43 |
| Analgesic drugs, n (%) | 85 (99) | 52 (99) | 1.00 |
| Antiepileptic drugs, n (%) | 10 (12) | 11 (21) | 0.15 |
| Leviracetam, n (%) | 10 (12) | 11 (21) | 0.15 |
| Valproate, n (%) | 4 (5) | 3 (6) | 1.00 |
|  |  |  |  |
| *Complications* |  |  |  |
| Stroke/ICH, n (%) | 14 (16) | 12 (23) | 0.38 |
| Brain death, n (%) | 14 (16) | 1 (2) | 0.01 |
| Bleeding, n (%) | 19 (22) | 14 (26) | 0.68 |
|  |  |  |  |
| *Outcome variables* |  |  |  |
| ICU stay, days | 8 [2-13] | 15 [8-31] | <0.01 |
| Hospital stay, days | 8 [2 -39] | 26 [9-63] | <0.01 |
| ICU death, n (%) | 58 (67) | 32 (60) | 0.47 |
| Hospital death, n (%) | 59 (69) | 32 (60) | 0.36 |
| GOS at 3 months | 1 [1-4] | 1 [1-4] | 0.40 |
| Poor neurological outcome, n (%) | 63 (73) | 36 (68) | 0.56 |
|  |  |  |  |
| *EEG findings* |  |  |  |
| Seizures/SE, n (%) | 4 (5) | 7 (13) | 0.10 |
| GPDs/LPDs, n (%) | 4 (5) | 6 (11) | 0.18 |
| Asymmetry, n (%) | 25 (17) | 14 (26) | 0.28 |
| Background Categories  *Mild/Moderate Encephalopathy, n (%)*  *Severe Encephalopathy, n (%)* | 51 (59)  15 (17) | 36 (68)  14 (26) | 0.04 |
| *Burst-Suppression, n (%)* | 4 (5) | 0 (0) |  |
| *Suppressed Background, n (%)* | 16 (19) | 3 (6) |  |

EEG= Electroencephalography; COPD= Chronic Obstructive Pulmonary Disease; NYHA= New York Heart Association; V-A ECMO = Veno-arterial Extracorporeal Membrane Oxygenation; V-V ECMO= Veno-venous Extracorporeal Membrane Oxygenation; RBC = Red Blood Cells; MAP = Mean Arterial Pressure; GCS = Glasgow Coma Scale; ICH = Intracranial Hemorrhage; ICU = Intensive Care Unit; GOS = Glasgow Outcome Scale; SE = Status Epilepticus; GPDs = Generalized Periodic Discharges; LPDs = Lateralized Periodic Discharges.
