# Supplementary material for: Electroencephalographic features in patients undergoing extracorporeal membrane oxygenation
Source: Crit Care. 2020 Oct 30;24:629. doi: 10.1186/s13054-020-03353-z (PMC7598240; doi:10.1186/s13054-020-03353-z)
Supplement: Supplementary file 10 — Additional file 10 Description of a case of early diagnose of stroke. [file 13054_2020_3353_MOESM10_ESM.docx]

**Additional File 10.**

**Supplemental Figure 1**. 73-year old male with V-A extracorporeal membrane oxygenation for cardiogenic shock.

1. Initial EEG recording showing diffuse low-voltage alpha and beta activity indicative of sedation.
2. EEG 5 days later showing diffuse slowing and left hemispheric attenuation.
3. Head computed tomography scan showing acute ischemic stroke in the left hemisphere and right parieto-occipital.

EEG settings : longitudinal bipolar montage ; low-pass filter 70Hz ; high-pass filter : 0.53 Hz ; notch filter off.


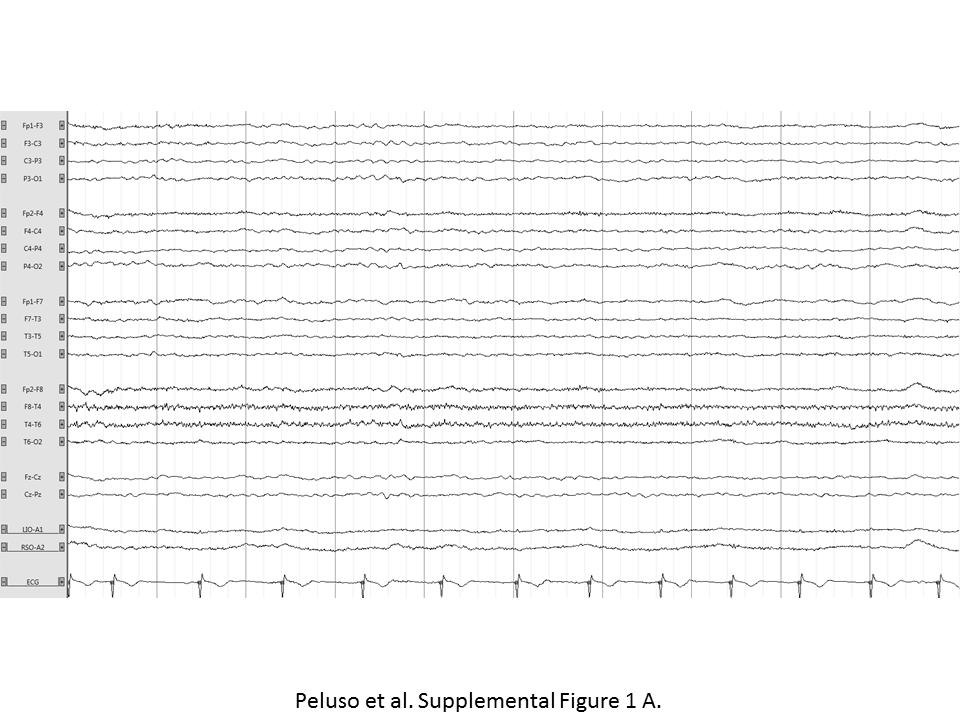


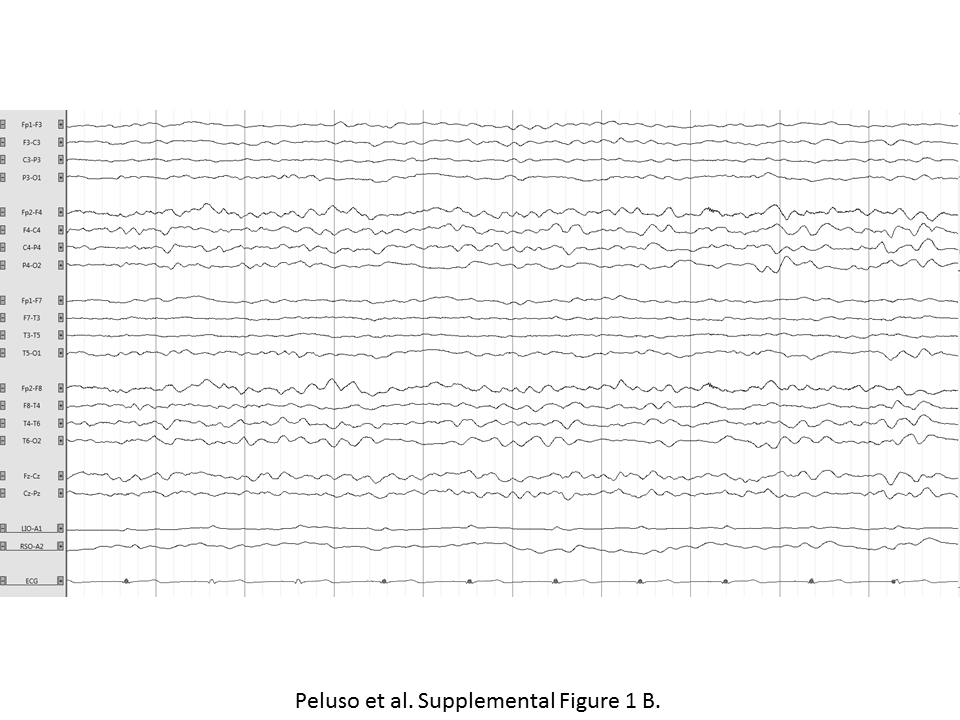


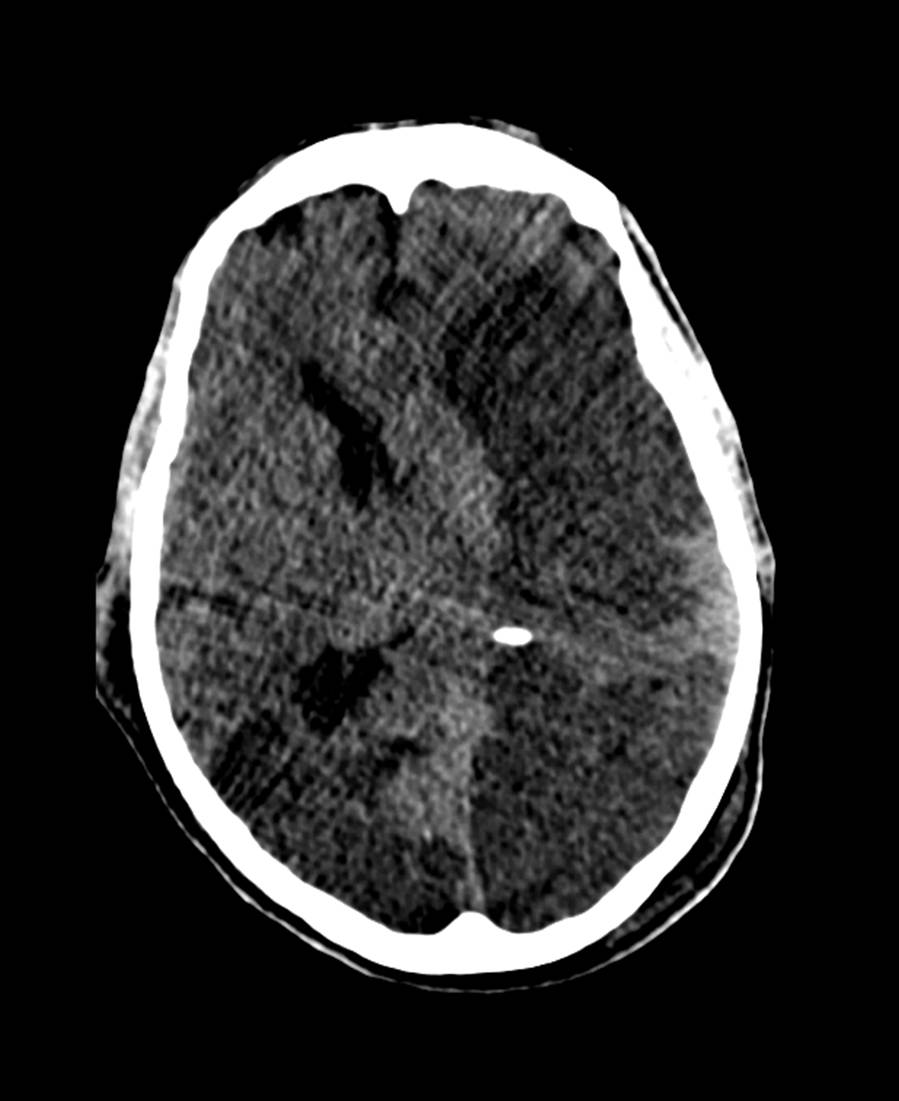


Peluso et al. Supplemental Figure 1C
