# Supplementary material for: Electroencephalographic features in patients undergoing extracorporeal membrane oxygenation
Source: Crit Care. 2020 Oct 30;24:629. doi: 10.1186/s13054-020-03353-z (PMC7598240; doi:10.1186/s13054-020-03353-z)

**Additional File 11**

**Supplemental Figure 2.** 60-year old female with V-A extracorporeal membrane oxygenation for cardiogenic shock.

1. Initial EEG showing generalized periodic discharges at 1 per second and right parasagittal rhythmic alpha activity, consistent with nonconvulsive status epilepticus.
2. Upon treatment with levetiracetam and midazolam, the EEG shows disappearance of ictal activity with abundant right fronto-temporal sharp waves. The patient ultimately presented a good neurological outcome at 3 months (GOS 5)

EEG settings : longitudinal bipolar montage ; low-pass filter 70Hz ; high-pass filter : 0.53 Hz ; notch filter off.


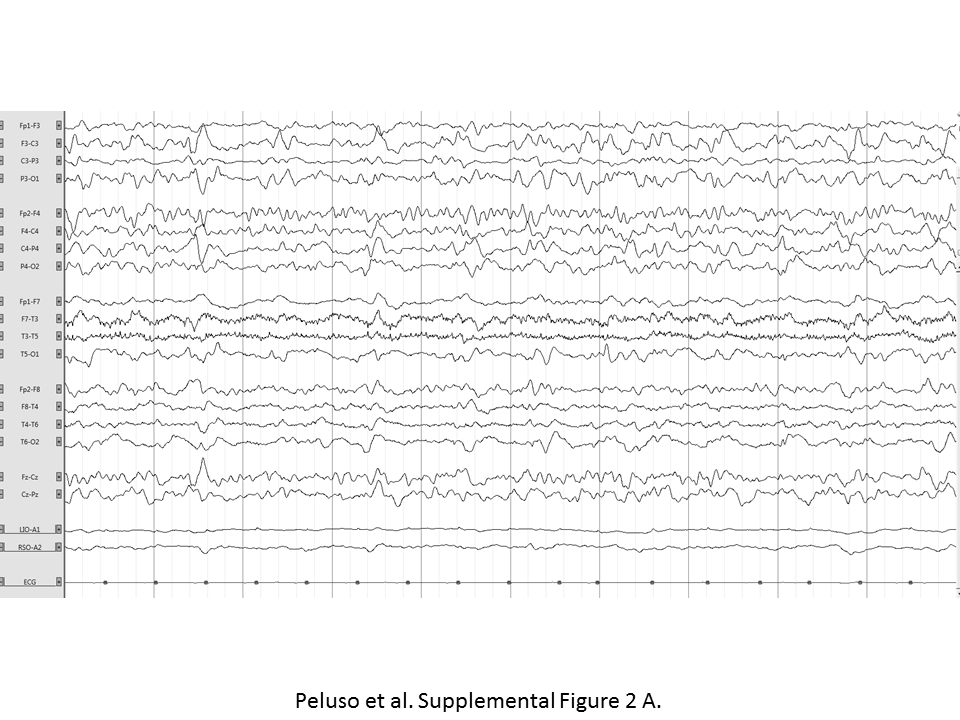


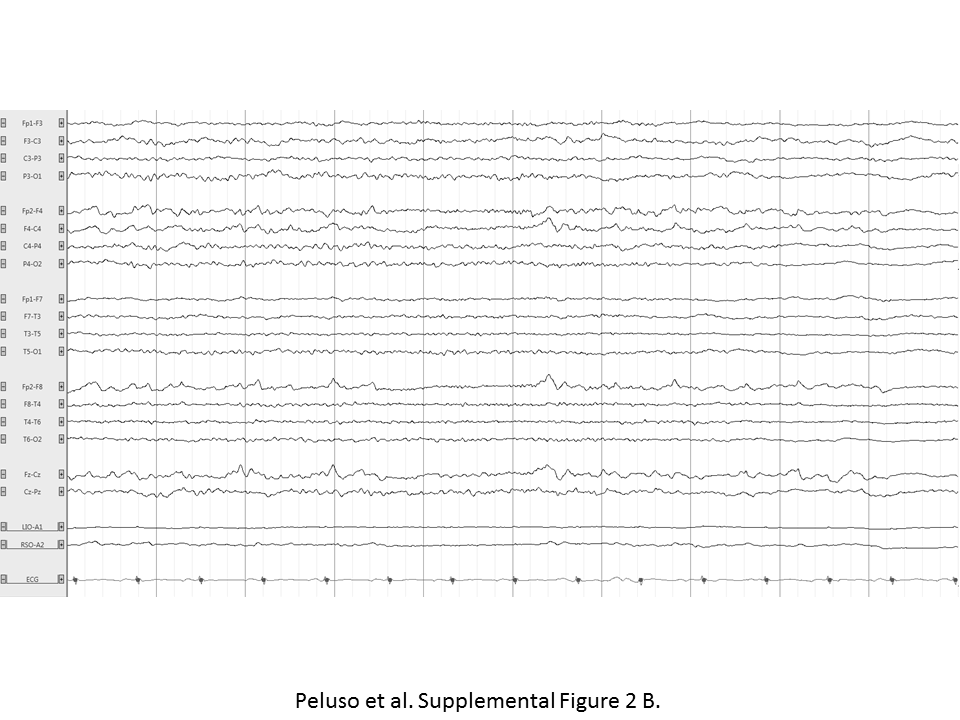

Supplement: Supplementary file 11 — Additional file 11 Description of a case of Nonconvulsive Status Epilepticus treated with success. [file 13054_2020_3353_MOESM11_ESM.docx]
